# Machine Learning Techniques for Predicting Drug-Related Side Effects: A Scoping Review

**DOI:** 10.3390/ph17060795

**Published:** 2024-06-17

**Authors:** Esmaeel Toni, Haleh Ayatollahi, Reza Abbaszadeh, Alireza Fotuhi Siahpirani

**Affiliations:** 1 Medical Informatics, Student Research Committee, Iran University of Medical Sciences, Tehran, Iran 14496-14535; toni.e@iums.ac.ir; 2 Medical Informatics, Health Management and Economics Research Center, Health Management Research Institute, Iran University of Medical Sciences, Tehran, Iran 1996-713883; 3 Pediatric Cardiology, Rajaie Cardiovascular Medical and Research Center, Iran University of Medical Sciences, Tehran, Iran 19956-14331; r.abbaszadeh@rhc.ac.ir; 4 Systems Biology and Bioinformatics, Department of Bioinformatics, Institute of Biochemistry and Biophysics (IBB), University of Tehran, Tehran, Iran 14176-14411; a.fotuhi@ut.ac.ir

**Keywords:** scoping review, machine learning, drug-related side effects, prediction

## Abstract

Background: Drug safety relies on advanced methods for timely and accurate prediction of side effects. To tackle this requirement, this scoping review examines machine-learning approaches for predicting drug-related side effects with a particular focus on chemical, biological, and phenotypical features. Methods: This was a scoping review in which a comprehensive search was conducted in various databases from 1 January 2013 to 31 December 2023. Results: The results showed the widespread use of Random Forest, k-nearest neighbor, and support vector machine algorithms. Ensemble methods, particularly random forest, emphasized the significance of integrating chemical and biological features in predicting drug-related side effects. Conclusions: This review article emphasized the significance of considering a variety of features, datasets, and machine learning algorithms for predicting drug-related side effects. Ensemble methods and Random Forest showed the best performance and combining chemical and biological features improved prediction. The results suggested that machine learning techniques have some potential to improve drug development and trials. Future work should focus on specific feature types, selection techniques, and graph-based methods for even better prediction.

## 1. Introduction

Drug-related side effects include undesirable, unpleasant, unexpected, and adverse hazardous reactions in organs and tissues [1]. Some market-approved drugs may cause unacceptable side effects, endangering human health and raising concerns among pharmaceutical companies [2]. Ensuring drug efficacy is crucial since unfavorable drug responses are the main cause of drug failure, often leading to side effects and drug withdrawal [2,3]. However, the traditional method of identifying side effects through solid clinical trials is time-consuming and expensive, making it unsuitable for large-scale tests [4,5]. As a result, there is a critical need to develop rapid and cost-effective methods for predicting drug-related side effects [6,7].

The ability to predict drug-related side effects presents itself as an indispensable facet of contemporary pharmaceutical research and development [8]. By enabling the early and accurate identification of potential side effects, such methodologies have the potential to revolutionize the drug development landscape, which can lead to significant time and resource efficiencies [9]. This transformative capacity facilitates the prioritization of drug candidates with favorable safety profiles while concurrently enabling the exclusion of those exhibiting a high propensity to induce adverse events [6,8]. Ultimately, the development of robust drug side effect prediction methodologies paves the way for the introduction of safer and more efficacious medications, thereby fostering improved patient outcomes and propelling advancements in personalized medicine [7,10,11].

The development of advanced computational algorithms provides strong technical support for addressing a wide range of medical challenges [12]. Specifically, numerous computational methods have been developed for predicting drug-related side effects, with a strong emphasis on machine learning-based approaches [13]. These methods delve into current information on drug-related side effects to create patterns that allow for the prediction of side effects for various drugs [1,13,14].

Recently, machine learning techniques emerged as the leading computational approaches for predicting drug-related side effects, leveraging previous experiences with similar drugs to learn and develop predictive models [1]. Existing machine learning-based approaches have rigorously examined hundreds of side effects and the probability of their occurrence [13,14]. This critical role of machine learning in side effect prediction entails developing models that predict outcomes based on the available data [1,15]. Machine learning techniques use drug properties and well-labeled side effects to predict drug-related side effects and build models for targeted predictions [16]. Integrating chemical, biological, and phenotypic features is critical in effectively predicting drug-related side effects, as diverse information and features from many sources contribute to the total understanding [17]. 

Researchers such as Pauwels et al. [18], Mizutani et al. [19], and Liu et al. [17] have contributed to the field by building drug-related side effect prediction models using various machine-learning techniques and incorporating different drug properties. Their findings highlight the importance of combining chemical, biological, and phenotypic data to make comprehensive drug-related side effect predictions [17,18,19]. Chemical features, such as molecular structure and composition, provide insights into a drug’s nature, while biological features explore interactions with cellular components [20,21]. Phenotypic features capture a drug’s effects on organisms, covering both therapeutic benefits and adverse reactions [22]. Integrating these features offers a holistic understanding of drug mechanisms and outcomes. Through machine learning analysis of these integrated features, robust predictive models can be developed, facilitating the early identification and mitigation of drug-related side effects [23,24]. These models empower researchers to optimize drug efficacy and safety profiles, ultimately leading to safer medications, improved patient outcomes, and advancing personalized medicine and pharmaceutical innovation [25]. 

Although several reviews have examined computational methodologies for predicting drug-related side effects, there are still significant gaps [1,13,14]. Das and Mazumder’s review of supervised machine-learning techniques looked at drug descriptors, commonly used drug property sources, and computational models, but they did not report or compare the performance of individual machine-learning algorithms [1]. Moreover, their focus did not encompass drug-related features. A separate review focused extensively on using computational techniques to predict drug-related side effects without comparing or comprehensively focusing on machine learning approaches [13]. Another review study examined three data sources, namely omics data, social network data, and electronic medical records, to predict adverse drug effects [14]. To our knowledge, none of the studies specifically focused on predicting drug-related side effects using drugs chemical, phenotypic, or biological features and machine learning techniques. Therefore, the aim of the current study was to review studies in which machine-learning techniques were used to predict drug-related side effects based on chemical, biological, or phenotypic features. 

## 2. Materials and Methods

This scoping review was conducted according to Arksey and O’Malley’s framework in 2023 [26]. Before conducting the research, ethics approval was obtained from the ethics committee of Iran University of Medical Sciences (IR.IUMS.REC.1401.1007).

### 2.1. Stage 1: Identifying Research Questions

A comprehensive understanding of machine learning techniques is essential to predict drug-related side effects based on chemical, biological, or phenotypic features for improving personalized medicine and safe medication prescriptions. Therefore, the research questions were as follows:

- What were the machine learning techniques used for predicting drug-related side effects?
- What were the main features used for predicting drug-related side effects?

### 2.2. Stage 2: Identifying Relevant Studies

The related articles were searched in different databases, including Web of Science, PubMed, Ovid, Scopus, ProQuest, IEEE Xplore, and the Cochrane Library. The search strategy included three main concepts: namely, “drug-related side effect”, “machine learning”, and “prediction”. The MeSH terms, synonyms, and other related keywords were also included in the search strategies. To identify the relevant papers, the search strategies were applied in three fields: title, abstract, and keywords of the articles (Supplementary Table S1). Articles were searched from 1 January 2013 to 31 December 2023. The citations and reference lists of the retrieved papers were also checked to ensure that all relevant studies were included.

### 2.3. Stage 3: Study Selection

In this study, the original research papers published in English between 2013 and 2023 with a focus on predicting drug-related side effects using chemical, biological, or phenotypical features were included. However, for papers that were published in languages other than English, there was no access to their full texts, review papers, letters to the editor, and papers that did not primarily focus on machine learning techniques were excluded.

The retrieved papers were entered into the Endnote software version 19, and after removing duplicates, the remaining articles were assessed in terms of the title and abstract relevancy to the study objective. After removing the irrelevant articles, the full texts of the remaining ones were examined by two authors (E.T. and H.A.) separately, and any disagreements were resolved by the third author (A.F.S.). 

### 2.4. Stage 4: Charting the Data

We used a data extraction form to collect the required data. This form contained the author’s name, publication year, country, study objective, selected features and data sources, algorithms, evaluation metrics, and main results. In this study, conducting a meta-analysis was not feasible due to the inherent heterogeneity of the study design and methodologies. As a result, the findings were organized and reported narratively. Regarding the evaluation metrics, including precision, accuracy, recall, F1 score, area under the curve (AUC), and area under the precision–recall curve (AUPR), the average was calculated and reported. 

## 3. Results

**Figure 1:**
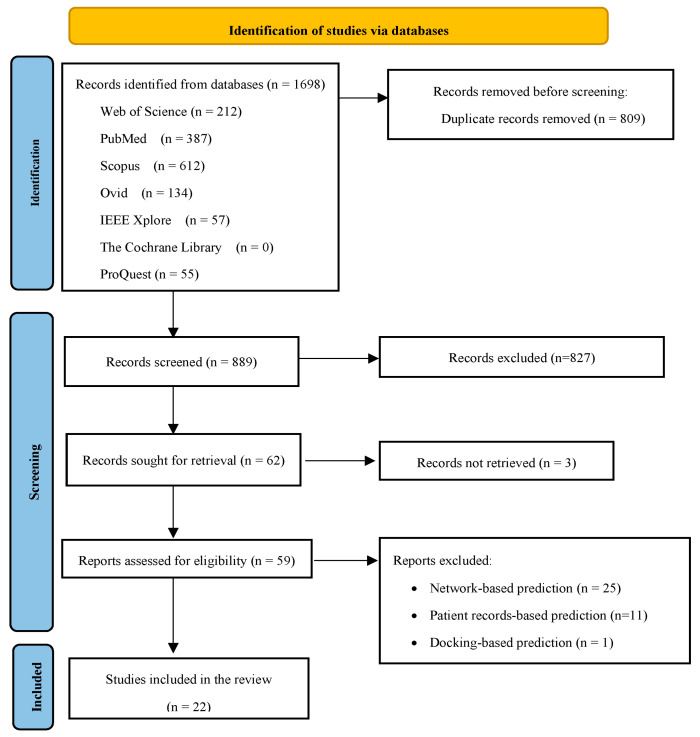
Selecting papers based on the PRISMA-ScR checklist [49].

In total, 1698 papers were retrieved from databases. After removing duplicates (n = 809), the remaining papers (n = 889) were examined in terms of their titles and abstracts, and irrelevant papers were excluded (n = 827). Among the remaining papers (n = 62), the full texts of three papers were not retrieved. As a result, the full texts of 59 papers were reviewed. Finally, 22 papers were selected to be included in the study [27,28,29,30,31,32,33,34,35,36,37,38,39,40,41,42,43,44,45,46,47,48]. A total of 37 papers were removed as either they were not related to machine learning algorithms or they did not include the expected features. The process of selecting the articles is illustrated in Figure 1.

### 3.1. Characteristics of the Selected Studies

The results showed that the highest frequency of the studies (n = 10) was conducted in China [27,28,32,37,39,40,44,45,46,47]. Other studies were completed in the United States (n = 3) [30,33,48], India (n = 2) [36,42], Turkey (n = 1) [29], Republic of Korea (n = 1) [31], Indonesia (n = 1) [34], Ireland (n = 1) [35], Australia (n = 1) [38], Taiwan (n = 1) [41], and the United Kingdom (n = 1) [43]. In 2017, the highest number of papers were published (n = 6) [38,39,40,41,42,43]. Table 1 shows a summary of the articles included in this study.

### 3.2. Selected Features and Data Sources

The study findings revealed that the selected features across various studies could be classified into four main categories, including general, chemical, biological, and phenotypical features. Different models employed one or more of these categories in predicting drug-related side effects. Furthermore, the data sources utilized for feature extraction displayed a degree of variability. DrugBank, Liu’s dataset, and SIDER 4 were consistently employed for extracting features across all categories. Bio2RDF v2 was utilized for all categories except for the general category, and Mizutani’s dataset was utilized across all categories except for the phenotypical category. The subsequent sections entail the features and data sources encompassed within each category.

#### 3.2.1. General Features

In most studies, general features containing drug generic names (n = 22) [27,28,29,30,31,32,33,34,35,36,37,38,39,40,41,42,43,44,45,46,47,48], drug-related side effects (n = 19) [27,29,30,32,33,34,35,36,38,39,40,41,42,43,44,45,46,47,48], and drug-related side effect pairs (n = 7) [28,30,37,38,39,47] were selected. These features were extracted from various sources, including SIDER (n = 17) [27,28,29,30,32,33,34,35,36,37,38,41,42,43,45,47], Pauwel’s dataset (n = 5) [40,44,45,46,48], Mizutani’s dataset (n = 5) [40,43,44,45,46], Liu’s dataset (n = 5) [40,43,44,45,46], DrugBank (n = 3) [31,39,41], Drug.com (n = 2) [29,39], OFFSIDES (n = 2) [27,33], and SIDER 4 (n = 2) [43,45]. Additionally, some studies extracted additional general features, including anatomical, therapeutic, and chemical (ATC) codes of drugs [28,30,32,33,37], drug categorization and frequency information [29,33], disease data [31], and the anatomical hierarchy of side effects [31]. These features were extracted respectively from the World Health Organization Collaborating Centre (WHOCC) [28,30,32,33,37], the Medical Dictionary for Regulatory Activities (MedDRA) [29,33], the Unified Medical Language System (UMLS) [31], and Wadhaw’s dataset [31].

#### 3.2.2. Chemical Features

Most studies extracted the chemical structure of drugs from diverse data repositories such as PubChem [29,31,32,36,41,42], Molecular Operating Environment [29], DrugBank [31,38,41], Bio2RDF v2 [35], SIDER 4 [35,43,45], Liu’s dataset [34,35,40,43,44,45,46], Pauwels’s dataset [40,44,45,46,47,48], and Mizutani’s dataset [40,43,44,45,46]. Also, some studies utilized established software tools like RDKit [27,28,30,33,37], ECFP_4 [30,37], Open Babel [41], and Tanimoto similarity tool [39] to extract associations between drug fingerprints using the simplified molecular input line entry specification (SMILES) strings. Moreover, some studies extracted drug structural association, drug literature association, and drug–drug interactions from SIMCOMP [28,30,37], STITCH [28,30,37], and STRING [31] databases, respectively. 

#### 3.2.3. Biological Features

Drug targets (n = 12) [29,34,35,36,40,41,42,43,44,45,46,47], transporters (n = 12) [29,34,35,36,40,41,42,43,44,45,46,47], enzymes (n = 11) [29,34,35,36,40,41,42,43,44,45,46], drug target-protein association (n = 6) [28,30,31,33,37,38], pathways (n = 4) [43,44,45,46], and carriers (n = 1) [41] were selected as biological features. These features were extracted from various sources, including DrugBank (n = 9) [28,29,30,31,33,36,37,41,42], Liu’s dataset (n = 7) [34,35,40,43,44,45,46], Pauwels’s dataset (n = 5) [40,44,45,46,47], Mizutani’s dataset (n = 5) [40,43,44,45,46], SIDER 4 (n = 3) [35,43,45], UniProt (n = 1) [41], EMBL-EBI (n = 1) [38], and Bio2RDF v2 (n = 1) [35]. 

#### 3.2.4. Phenotypical Features

The most selected phenotypical features were therapeutic indications (n = 10) [13,14,15,19,20,21,22,23,24,25]. These features were extracted from various sources, including SIDER 4 [35,43,45], Liu’s dataset [34,35,40,43,44,45,46], SIDER [36,42], DrugBank [41], National Drug File-Reference Terminology (NDF-RT) [41], Bio2RDF v2 [35]. Other phenotypical features, including cell culture [30], single nucleotide polymorphism [31], and other ADRs [36,42], were extracted from the National Cancer Institute, DisGeNET, and SIDER, respectively.

### 3.3. Algorithms and Evaluation Metrics

The primary findings indicated that the most frequently employed algorithms in studies were Random Forest (n = 8, 36%) [28,30,31,35,36,37,40,41], k-nearest neighbor (KNN) (n = 7, 31%) [28,34,35,38,39,41,46], support vector machine (SVM) (n = 6, 27%) [27,28,32,38,39,47], multi-layer perceptron (MLP) (n = 5, 22%) [28,29,34,35,47], naive Bayes (n = 3, 13%) [28,31,41], and logistic regression (n = 3, 13%) [27,28,31]. Additionally, the results revealed that AUC (n = 19, 86%) [27,28,29,30,31,32,33,34,35,36,40,41,42,43,44,45,46,47,48], F1 score (n = 12, 54%) [27,28,29,31,34,36,37,38,39,41,42,44], precision (n = 11, 50%) [27,30,31,32,35,36,39,42,44,45,46], recall (n = 10, 45%) [27,28,30,31,36,37,39,41,42,44], AUPR (n = 10, 45%) [28,30,32,35,40,43,44,45,46,47], and accuracy (n = 8, 36%) [28,32,34,36,41,42,44,48] were the predominantly utilized evaluation metrics.

### 3.4. Comparison of Evaluation Metrics

The results showed that Random Forest was the best machine-learning algorithm. It had the highest AUC (0.97) [36], precision (0.94) [31], recall (0.98) [31], F1 Score (0.97) [31], and AUPR (0.977) [23,31] among other algorithms. SVM (0.95) [27] and integrated neighborhood-based method (INBM) (0.959) [39] had the highest rate of accuracy. Table 2 contains detailed information on each algorithm’s performance.

According to Table 2, ensemble methods [27,28,30,31,35,36,37,40,41,48] demonstrated superior performance with an average precision of 0.78 and an AUPR of 0.76. SVM methods [27,28,32,36,38,42,47] excelled with an average accuracy of 0.94 and a recall of 0.89. Decision trees [28,35] displayed strong performance with an average F1 score of 0.91, while clustering methods [43] showcased notable performance with an average AUC of 0.89.

The results revealed that different combinations of chemical, biological, and phenotypical have an impact on evaluation metrics. Based on this study, the integration of both chemical and biological features yielded the highest performance across all algorithms, as evidenced by precision (0.79), F1 score (0.85), AUC (0.83), and AUPR (0.579) [28,29,33,36,37,38,40,41,47]. Moreover, the combination of chemical, biological, and phenotypical features demonstrated superior average accuracy (0.93) across all algorithms [30,31,34,35,36,38,40,41,42,43,44,45,46]. Additionally, employing solely chemical features [27,32,36,39] outperformed other combinations, resulting in an average recall of 0.83. 

## 4. Discussion

### 4.1. Principal Findings

This scoping review investigated the use of machine learning techniques for the prediction of drug-related side effects. Based on the findings, general features were mainly extracted from SIDER, Pauwel’s dataset, Mizutani’s dataset, Liu’s dataset, and DrugBank. Chemical features predominantly were obtained from PubChem, Molecular Operating Environment, and DrugBank using fingerprint analysis software. DrugBank, Liu’s dataset, and Pauwels’ dataset were used to provide biological features, and SIDER 4, Liu’s dataset, SIDER, DrugBank, and Bio2RDF v2 provided therapeutic indications and phenotypes.

According to the current review findings, when chemical and biological features were combined, the prediction outcomes were impressive. Moreover, ensemble methods showed the best results in terms of precision and AURP metrics. SVM exhibited superior performance in accuracy and recall measures, and decision trees excelled in F1 score metrics. In addition, clustering methods demonstrated proficiency in AUC assessment.

The results showed that careful selection of features from relevant databases or datasets is crucial in predicting drug-related side effects. In the present study, features were classified into four primary groups. This classification scheme is aligned with the findings reported by Das and Mazumder’s study [1]. Likewise, the review conducted by Sachdev and Gupta on computational techniques for identifying drug-related side effects introduced some features and datasets [13]; however, the focus was not primarily on machine learning techniques, resulting in a limited range of features compared to the current study. 

Various studies highlighted the importance of specific features in predicting drug-related side effects, such as chemical fingerprints from SMILES strings and target protein associations from DrugBank, indicating the necessity for a combination of chemical and biological data for accurate predictions. However, biases exist within data sources like SIDER, which may skew towards common side effects [50], and limitations in PubChem exclude information on biologic drugs, urging integration with databases capturing biologic complexities [51]. Feature engineering techniques, like fingerprint generation algorithms and text-mining, aid in translating raw data into interpretable formats [52], while network-based approaches offer promise in modeling complex relationships between chemical structures, biological targets, and side effects [53]. Despite the potential of emerging data sources such as electronic health records and genomics data for personalized prediction, challenges like data standardization and interoperability persist [54], highlighting the need for standardized efforts and common ontologies to facilitate comprehensive dataset creation for machine learning models in side effect prediction.

According to the findings of this review, the integration of chemical and biological features showcased proficiency in precision, F1 score, AUC, and AUPR metrics. In the research conducted by Mizutani et al., canonical correlation analysis and sparse canonical correlation analysis were used, which provided valuable insights into the significance of feature selection. Their study highlighted the superiority of employing the targeted protein-based approach as a biological feature for the prediction of drug-related side effects [19]. Moreover, the research conducted by Liu et al. evaluated various machine-learning algorithms by different features and demonstrated the exceptional performance of SVM when combining chemical, biological, and phenotypic features [17].

Random Forest emerged as the most common algorithm used across the included studies, followed by KNN and SVM. However, there are discrepancies regarding the most frequently used algorithms within this research domain [55]. Das and Mazumder reported that SVM and logistic regression are commonly used for predicting drug-related side effects [1]. In contrast, Sachdev and Gupta emphasized the efficacy of multi-label KNN learning, SVM, and random forest [13]. Random Forest interpretability and resistance to overfitting are among the advantages of this algorithm; however, it may struggle with high-dimensional data [56]. Techniques like Mean Decrease in Impurity (MDI) could enhance its efficacy [57]. KNN is valued for simplicity but requires careful parameter selection, while SVM handles high-dimensional data well but can be computationally expensive [58]. Beyond these, gradient-boosting machines and deep learning architectures offer promising alternatives and are adept at capturing complex relationships in drug data [6].

This study highlighted the significance of different feature combinations in predicting outcomes. Similarly, Das and Mazumder focused on four distinct features, namely, chemical, biological, phenotypic, and other drug descriptors [1]. Other studies concentrated on patient-centric data sources such as prospective data collection and derived data from Electronic Health Records (EHRs) and social media platforms to enrich their predictive capabilities [59]. For example, Zhao et al. used EHR data to predict drug-related side effects. They applied multiple supervised algorithms to analyze patient data, including demographics, lab results, and medication history, achieving significant accuracy with the Random Forest algorithm in identifying potential drug-related side effects before they manifested clinically [60]. Ietswaart et al. used data from the FDA’s Adverse Event Reporting System (FAERS) to train a Random Forest model. This model was able to detect subtle patterns and correlations within the vast datasets, effectively predicting the side effects of new and existing drugs [61]. 

It is essential to distinguish between studies that used patient-centric data and those that focused on drug features, as their objectives vary significantly. Patient-centric studies primarily aim to predict the overall incidence of specific drug-related side effects, diagnose individuals experiencing side effects, or prognosticate patients at high risk of drug side effects [62]. Conversely, studies included in this review predominantly focused on predicting drug-related side effects based on drug features prior to their manifestation in patients. For instance, Kim et al. reviewed existing statistical and machine-learning methods to detect drug-related side effects in humans [59]. La et al. integrated theoretical biological data into machine-learning models to predict Active Pharmaceutical Ingredient (API) side effects, validating their approach against real-world clinical outcomes [63]. This underscores the multifaceted nature of data used in predicting drug-related side effects, reflecting the inherent challenges in directly comparing machine learning techniques used across these two distinct groups of studies.

Additionally, different metrics, including AUC, F1 score, precision, recall, AUPR, and accuracy, were used to evaluate the algorithm’s effectiveness. According to the results, AUC was the most frequently used metric. These findings are consistent with Ho et al., who underscored the importance of metrics such as AUC, F1 score, and precision in evaluating machine learning algorithms for ADR detection and prediction [14]. Drug-related side effect data often suffer from class imbalance, where some side effects are significantly rarer than others [33]. Exploring alternative metrics like balanced accuracy or Matthew’s correlation coefficient, which accounts for class imbalance, could provide a more nuanced perspective on model performance, especially for datasets with imbalanced classes [64].

The results showed that Random Forest had superior performance compared to other machine learning algorithms included in this study. However, the prominent algorithm in Das and Mazumder’s study was SVM [1], and multi-label KNN learning prevailed in Sachdev and Gupta’s research [13]. Random Forest’s prominence in drug-related side effect prediction arises from its adeptness at handling high-dimensional data and its robustness to imbalanced class distributions commonly found in such datasets [9]. Ensemble methods like Random Forest often outperform single-learner methods like SVM due to their ability to leverage multiple learners for greater generalizability, although SVMs may excel in specific scenarios, particularly with limited dataset sizes [36]. However, a deeper analysis beyond average performance metrics is essential to unveil algorithm-specific nuances and assess generalizability across independent datasets [58]. Combining chemical and biological features enhances performance, but further exploration into specific types of features and feature selection techniques is warranted. 

Overall, a comprehensive examination of multiple studies reveals common trends and variations in the selection of features, databases, and algorithms for predicting drug-related side effects. The diversity of machine learning approaches highlighted the complex nature of this task, and the emphasis on using different evaluation metrics underscores the significance of thorough evaluation to guarantee the reliability and effectiveness of predictive models in the pharmaceutical research domain.

### 4.2. Implication for Practice

By leveraging comprehensive datasets that integrate chemical, biological, and phenotypic information, machine learning algorithms demonstrate promise in robustly predicting drug-related side effects. These capabilities translate into several key benefits for clinical translation and drug development applications. This type of prediction can facilitate a paradigm shift towards precision medicine. Integration of pharmacogenetic data into these algorithms could empower clinicians to tailor drug therapies based on the individual patient’s unique genetic profiles, significantly mitigating the risk of drug-related side effects [25,65]. Furthermore, machine learning can serve as a powerful catalyst in drug development by enabling the early identification of potential side effects. This crucial information allows researchers to prioritize promising drug candidates and circumvent late-stage clinical trial failures stemming from unforeseen safety concerns [66].

Machine learning also offers the potential to optimize clinical trial design through patient stratification based on the risk of the predicted side effect. This targeted approach can enhance the efficiency and safety of clinical trials by focusing on patient populations demonstrably susceptible to side effects [67]. Additionally, graph learning approaches have emerged as a powerful tool for uncovering the intricate relationships between drugs, their targets, and potential side effects [68,69,70]. By leveraging biological networks that integrate information on drugs, targets, and their interactions, graph neural networks (GNNs) offer a promising avenue for improved prediction [71]. However, GNN-based methods are susceptible to the over-smoothing problem, which can hinder their ability to learn discriminative representations of drugs and targets [72]. To address this challenge, recent studies have proposed novel GNN architectures that incorporate strategies to mitigate over-smoothing, such as node-dependent local smoothing techniques [73]. These advancements pave the way for more accurate drug side effect predictions by capturing the nuanced relationships within biological networks [71,72].

### 4.3. Strengths and Limitations of the Study

In this study, the literature related to the use of machine learning algorithms for predicting drug-related side effects, their selected features, and evaluation metrics were reviewed. However, there were some limitations. First of all, due to the inherent diversity in the design, datasets, and methodologies across the literature, conducting a meta-analysis was not feasible. In response to this limitation, a qualitative comparison approach was adopted, enabling a comprehensive evaluation of the available evidence. The second limitation was related to the exclusion of non-English studies due to time and resource constraints. The third limitation might be related to overfitting the included models, particularly as they are in-silico models lacking empirical verification in real-world scenarios. To mitigate this concern, future research can prioritize the validation of predictive models using real-world data and clinical trials. Rigorous cross-validation techniques and external validation on independent datasets can further enhance the robustness and generalizability of predictive models. Moreover, the algorithms should be used to predict the side effects of the commercially available drugs to be able to evaluate their performance and effectiveness.

## 5. Conclusions

In conclusion, this scoping review comprehensively analyzed the use of machine learning techniques for predicting drug-related side effects. The findings underscore the critical role of selecting features from diverse databases encompassing chemical, biological, and phenotypic data for robust prediction. Ensemble methods, particularly Random Forest, emerged as superior algorithms across a spectrum of evaluation metrics, including AUC, precision, recall, F1 score, and AUPR. To predict drug-related side effects, the integration of chemical and biological features enhanced performance. These findings suggested that machine learning algorithms are useful for various applications in the pharmaceutical domain, including drug development through early prediction of side effects and optimizing clinical trial designs via patient stratification based on the predicted risk of side effects. Future research should delve into exploring specific feature types, refining feature selection techniques, and investigating the potential of graph-based methods to predict even more accurate drug-related side effects.

## Figures and Tables

**Table 1:** Summary of the selected articles.

No	Author, Year, and Country	Objective	Selected Features and Data Sources				Algorithm(s)	Evaluation Metrics	Main Results
			General Features	Chemical Features	Biological Features	Phenotypical Features			
1	Chen et al., 2023 [27], China	To predict adverse drug reactions and mining importance features based on the SIDER dataset	Drugs and drug–side effects Data source: SIDER, OFFSIDES	Drug fingerprint association Software: RDKit	_	_	Logistic regression, SVM, XGBoost, AdaBoost, single-layer stacking, multi-layer stacking	F1 score, AUC, recall, precision	Single-layer stacking, on average, had the highest AUC of 0/684 SVM, on average, had the highest recall of 0/92 Multi-layer stacking, on average, had the highest precision of 0/735 and F1 score of 0/696.
2	Wu and Chen., 2022 [28], China	To predict drug side effects using similarity-based multiple-feature sampling.	▪ Drugs (n = 841), drug–side effect pairs (n = 57,058) Data source: SIDER ▪ Drug ATC code	▪ Drug fingerprint association Software: RDKit ▪ Drug structural association Data source: SIMCOMP ▪ Drug literature association Data source: STITCH	Drug target protein association Data source: DrugBank	_	SVM, Adaboost, Random Forest, Bayesian network, naive Bayes, KNN, decision tree, PART, logistic regression, MLP, RIPPER	Recall, specificity, accuracy, MCC, F1 score, AUC, AUPR	Random Forest had the highest performance with MCC of 0.8661, AUC of 0.969, and AUPR of 0.977.
3	Güneş et al., 2021 [29], Turkey	To predict adverse drug reactions in a predictive model by integrating drug chemical structures and biological properties.	▪ Drugs (n = 27) ▪ Drugs’ categorization and frequency information Data sources: MedDRA, Drugs.com, SIDER ▪ Drug–side effect (n = 329) Data source: SIDER	Chemical structure information Data sources: PubChem, Molecular Operating Environment	Biological properties (i.e., targets, enzymes, and transporters) Data source: DrugBank	_	MLP	MCC, F1 score, AUC, NPV	MLP had a high performance with both chemical and biological features (AUC = 0.695)
4	Zhou et al., 2020 [30], USA	To use machine learning for predicting small molecule drug side effects, indications, efficacy, and mode of action.	▪ Drugs (n = 841), drug–side effect (n = 824), drug–side effect pairs (n = 57,058) ▪ Data source: SIDER ▪ Drug ATC code	▪ Drug fingerprint association Software: RDKit, ECFP_4 ▪ Drug structural association Data source: SIMCOMP ▪ Drug literature association Data source: STITCH	Drug target protein association Data source: DrugBank	Cell culture Data source: National Cancer Institute/Division of Cancer Treatment and Diagnosis/Developmental Therapeutics Program	Random Forest	AUPR, AUC, recall, precision	Random Forest had a high performance (AUC = 0.902).
5	Seo et al., 2020 [31], Republic of Korea	To propose a machine learning approach to predict potential drug side effects.	▪ Drugs (n = 2144) Data source: DrugBank ▪ Side effect anatomical hierarchy Data source: Wadhaw et al. ▪ Disease (n= 6556) ▪ Data source: UMLS	▪ Drug-drug interactions Data sources: Drug Bank, STRING ▪ Chemical structure ▪ Data sources: DrugBank, PubChem	Drug target protein interaction Data sources: DrugBank, Therapeutic Target Database	Single nucleotide polymorphism Data sources: Comparative Toxicogenomic Database, DisGeNET	Naive Bayes, logistic regression, Random Forest, XGBoost	Recall, precision, specificity, F1 score, AUC	Random Forest and XGBoost had the highest precision Random Forest had the highest performance with a specificity of 0.8126, precision of 0.8178, recall of 0.8473, and F1 score of 0.8294.
6	Jiang et al., 2020 [32], China	To propose a preliminary machine learning model for drug side-effects prediction	▪ Drugs (n = 888), drug–side effect (n = 1385) Data source: SIDER ▪ Drug ATC code	Chemical structure ▪ Data source: PubChem	_	_	SVM, regularized regression, LNSM	AUC, AUPR, accuracy, hamming loss, ranking loss, one error, coverage, average precision	The KNN model has a higher average accuracy (95.37%) than other methods. SVM (WGTS kernel) had the highest performance with an AUC of 0.9052 The LNSM model had the highest performance with an AUPR of 0.4491
7	Galeano et al., 2020 [33], USA	To propose a machine learning framework for drug side effect frequency prediction.	▪ Drugs (n = 759), drug–side effect (n = 994) Data sources: SIDER, OFFSIDES ▪ Drugs’ frequency information Data sources: MedDRA, SIDER ▪ Drug ATC code	Drug fingerprint association ▪ Software: RDKit	Drug target protein interaction Data source: DrugBank	_	Matrix decomposition model	RMSE, AUC	The drug side effect model predicted Semagacestat side effect frequencies in phases 2 and 3 with AUC values of 0.853 and 0.810. The model predicted drug-shared protein targets with 68.38% AUC action biology.
8	Afdhal et al., 2020 [34], Indonesia	To use multi-label linear discriminant analysis and learning for predicting adverse drug reactions	▪ Drugs (n = 832), drug–side effect (n = 626) ▪ Data source: SIDER	Chemical substructure ▪ Data sources: Liu’s dataset	Drug targets, transporters, enzymes Data sources: Liu’s dataset	Indications Data sources: Liu’s dataset	KNN, MLP	AUC, Hamming loss, F1 score, accuracy	KNN and MLP algorithms increase AUC by 1.56% and 6.53%, respectively.
9	Muñoz et al., 2019 [35], Ireland	To predict adverse drug reactions using knowledge graphs and multi-label learning models	▪ Drugs (n = 832), drug–side effect (n = 626) ▪ Data source: SIDER	Chemical substructure ▪ Data sources: Liu’s dataset, Bio2RDF v2, SIDER 4	Drug targets, transporters, enzymes Data sources: Liu’s dataset, Bio2RDF v2, SIDER 4	Indications Data sources: Liu’s dataset, Bio2RDF v2, SIDER 4	KNN, decision tree, linear regression, MLP, Random Forest	Average precision, AUPR, AUC, ranking loss, one-error, coverage	According to average precision and AUPR metrics, Bio2RDF v2 performed slightly better with Liu’s dataset. MLP outperformed other models in AUC, ranking-loss, and one-error metrics.
10	Jamal et al., 2019 [36], India	To propose a machine learning-based exhaustive computational model for adverse cardiovascular drug reaction prediction.	Drugs (n = 965), side effects ▪ Data source: SIDER	Chemical structure (n = 881) ▪ Data source: PubChem	Drug targets (n = 1264), transporters (n = 86), enzymes (n = 182) Data source: DrugBank	Therapeutic indications (n = 1840), Other ADRs (n = 5497) Data source: SIDER	SMO, Random Forest	Recall, precision, accuracy, specificity, F1 score, AUC	Integrating biological, chemical, and phenotypic features increased random forest and SMO model AUC values. Phenotypical features increased accuracy values, demonstrating their potential to improve model predictiveness.
11	Zhao et al., 2018 [37], China	To propose a similarity-based method for drug side effects prediction with heterogeneous information.	▪ Drugs (n = 841), drug–side effect pairs (n = 57,058) Data source: SIDER Drug ATC code	▪ Drug fingerprint association Software: RDKit, ECFP_4 ▪ Drug structural association Data source: SIMCOMP ▪ Drug literature association Data source: STITCH	Drug target protein association Data source: DrugBank	_	Random Forest	Recall, specificity, accuracy, MCC, F1 score	The model had an average sensitivity of 0.791, specificity of 0.759, accuracy of 0.775, precision of 0.766, and F1-measure 0.778. The model predicted drug side effects effectively with an MCC of 0.8492.
12	Zheng et al., 2017 [38], Australia	To improve side-effect prediction with an optimized drug similarity framework.	Drugs (n = 917), side effect (n = 500), drug–side effect pairs (n = 78,855) Data source: SIDER	Drug substituent, therapeutic, chemical structure similarity Data source: DrugBank	Drug target protein similarity Data source: EMBL-EBI website	_	KNN, SVM, ELM, RBF	F1 score	SVM had the highest side-effect prediction performance improvement (18.4%), and KNN had the lowest (up to 2.5%) in F1 scores.
13	Sun et al., 2017 [39], China	To predict drug side effects using drug similarities and known side effects.	Drugs (n = 1134), side effect (n = 300), drug–side effect pairs (n = 75,578) Data source: DrugBank, DrugCom	Drug chemical formula similarity: Software: Tanimoto similarity tool from CDK v1.5.13	_	_	KNN, SVM	Recall, precision, F1 score	The comprehensive similarity-based approach using KNN outperformed SVM-based methods with an average F1-Score of 70.91%, recall of 92.80%, and precision of 57.57%.
14	Niu and Zhang., 2017 [40], China	To quantitatively predict drug side effects using drug features.	Drugs (n = 888), side effects (n = 1385) Data sources: Pauwels’s dataset, Mizutani’s dataset, Liu’s dataset	Chemical substructure Data sources: Pauwels’s dataset, Mizutani’s dataset, Liu’s dataset	Drug targets, transporters, enzymes Data sources: Pauwels’s dataset, Mizutani’s dataset, Liu’s dataset	Indications Data sources: Liu’s dataset	Random Forest	AUPR, AUC, RMSE, R^2^	In Pauwels’s, Mizutani’s, and Liu’s datasets, Random Forest algorithms had AUPR scores of 0.2509 to 0.4117, AUC scores of 0.9, RMSE values of 0.0390 to 0.0496, and R2 values of 0.0237 to 0.2893.
15	Lee et al., 2017 [41], Taiwan	To propose a hybrid machine learning approach to create side effect predictors using relevant data features.	Drugs (n = 1002), side effects (n = 3903) Data source: DrugBank, SIDER	Chemical substructure Data sources: DrugBank, PubChem Software: Open Babel	Drug targets, transporters, enzymes, carrier Data sources: DrugBank, UniProt	Indications Data sources: DrugBank, ND-FRT	Native Bayes, KNN, Random Forest	Recall, specificity, accuracy, AUC, F1 score	The Native Bayes algorithm had an AUC of 0.89 and an F1 score of 0.81, the KNN algorithm had 0.87 and 0.80, and the Random Forest algorithm had 0.90 and 0.85.
16	Jamal et al., 2017 [42], India	Use machine learning models based on drug biological, chemical, and phenotypic properties to predict neurological adverse drug reactions.	Drugs (n = 965), side effects Data source: SIDER	Chemical structure (n = 881) Data source: PubChem	Drug targets (n = 1264), transporters (n = 86), enzymes (n = 182) Data source: DrugBank	Therapeutic indications (n = 1840), Other ADRs (n = 5497) Data source: SIDER	SMO	Recall, precision, accuracy, F1 score, AUC	Chemical + phenotypic properties models predicted neurological adverse drug reactions better than models based on individual properties or their combinations, with the highest F1 score and AUC of 0.96.
17	Dimitri and Lió., 2017 [43], UK	To demonstrate a drug side effect prediction by machine learning algorithm.	Drugs (n = 888), side effects (n = 1385) Data sources: SIDER 4, Mizutani’s dataset, Liu’s dataset	Chemical substructure Data sources: SIDER 4, Mizutani’s dataset, Liu’s dataset	Drug targets, transporters, enzymes, pathway Data sources: SIDER 4, Mizutani’s dataset, Liu’s dataset	Indications Data sources: SIDER 4, Liu’s dataset	K-means, PAM, K-seeds	AUC, AUPR	AUC performance for protein targets, chemical substructure, and their combination is consistently higher for K-seeds than K-means and PAM across all three datasets.
18	Zhang et al., 2016 [44], China	Use approved drugs, side effect terms, and drug–side effect associations to create a recommender system for side effect prediction.	Drugs (n = 888), side effects (n = 1385) Data sources: Pauwels’s dataset, Mizutani’s dataset, Liu’s dataset	Chemical substructure Data sources: Pauwels’s dataset, Mizutani’s dataset, Liu’s dataset	Drug targets, transporters, enzymes, pathway Data sources: Pauwels’s dataset, Mizutani’s dataset, Liu’s dataset	Indications Data sources: Liu’s dataset	INBM, RBMBM	Recall, precision, accuracy, F1 score, AUC, AUPR	In all three datasets, RBMBM had higher AUC scores than INBM, suggesting it may be better at drug side effect prediction. INBM still had competitive AUC scores and may be a good alternative.
19	Zhang et al., 2016 [45], China	To predict drug side effects using linear neighborhoods and integrating multiple data sources.	Drugs (n = 888), side effects (n = 1385) Data sources: SIDER 4, Pauwels’s dataset, Mizutani’s dataset, Liu’s dataset	Chemical substructure Data sources: SIDER 4, Pauwels’s dataset, Mizutani’s dataset, Liu’s dataset	Drug targets, transporters, enzymes, pathway Data sources: SIDER 4, Pauwels’s dataset, Mizutani’s dataset, Liu’s dataset	Indications Data sources: SIDER 4, Liu’s dataset	LNSM	Average precision, AUPR, AUC, ranking loss, Hamming loss, one-error, coverage	The AUC values for the LNSM are 0.8941 for the SIDER 4 dataset, 0.8941 for Pauwels’s dataset, 0.8946 for Mizutani’s dataset, and 0.8850 for Liu’s dataset.
20	Zhang et al., 2015 [46], China	Drug side effect prediction using multi-label and ensemble learning.	Drugs (n = 888), side effects (n = 1385) Data sources: Pauwels’s dataset, Mizutani’s dataset, Liu’s dataset	Chemical substructure Data sources: Pauwels’s dataset, Mizutani’s dataset, Liu’s dataset	Drug targets, transporters, enzymes, pathway Data sources: Pauwels’s dataset, Mizutani’s dataset, Liu’s dataset	Indications Data sources: Liu’s dataset	FS-MLKNN, MLKNN	Average precision, AUPR, AUC, ranking loss, Hamming loss, one-error, coverage	FS-MLKNN outperformed MLKNN in all features tested, including chemical substructures, drug targets, transporters, enzymes, pathways, and indications.
21	Niu et al., 2015 [47], China	To develop a novel method to predict potential adverse drug reactions based on chemical substructures.	Drugs (n = 697), side effect (n = 2604), drug–side effect pairs (n = 74,343) Data source: SIDER	Chemical substructure Data source: Pauwels’s dataset	Drug targets and transporters Data source: Pauwels’s dataset	_	MLP, SVM, kernel regression, sparse canonical correlation analysis	AUPR, AUC	The highest AUC score was 0.8927 for MLP, followed by SVM (0.7984), sparse canonical correlation analysis (0.8811), and kernel regression (0.8576).
22	Jahid and Ruan., 2013 [48], USA	To propose a chemical structure-based ensemble model to predict drug side effects.	Drugs (n = 888), side effects (n = 1385) Data source: Pauwels’s dataset	Chemical substructure Data source: Pauwels’s dataset	_	_	Multi-layer staking	AUC, accuracy	With an average AUC of 0.87, the model predicted 1032 out of 1385 side-effect terms for drug molecules with 0.77 accuracy.

Abbreviations: AdaBoost (Adaptive Boosting); ATC (Anatomical Therapeutic Chemical); AUC (Area under the Curve); AUPR (Area Under the Precision–Recall curve); FS-MLKNN (Feature Selection-based Multi-label K-Nearest Neighbor); INBM (Integrated Neighborhood-Based Method); KNN (K-Nearest Neighbor); LNSM (Linear Neighborhood Similarity Method); MCC (Matthews Correlation Coefficient); MedDRA (Medical Dictionary for Regulatory Activities); MLKNN (Multi-label K-Nearest Neighbor); MLP (Multi-layer Perceptron); NDF-RT (National Drug File-Reference Terminology); NPV (Negative Predictive Value); PAM (Partition Around Medoids); RBF (Radial Basis Function); RBMBM (Restricted Boltzmann Machine-Based Method); RIPPER (Repeated Incremental Pruning to Produce Error Reduction); RMSE (Root Mean Squared Error); SIMCOMP (SIMilar COMPound); SMO (Sequential Minimal Optimization); SVM (Support Vector Machine); UMLS (Unified Medical Language System); XGBoost (eXtreme Gradient Boosting).

**Table 2:** Comparing algorithms, selected features, and evaluation metrics.

Algorithm (Number of Studies)		Selected Features			Evaluation Metrics						Ref.
		Chemical	Biological	Phenotypical	Precision	Accuracy	Recall	F1 Score	AUC	AUPR	
Ensemble methods	Random Forest (n = 8)	✓			0.94	0.8875	0.97	0.95	0.52	0.95	[36]
		✓	✓		0.8860	0.8944	0.8201	0.8514	0.969	0.977	[28]
					0.94	0.9381	0.98	0.96	0.55	0.95	[36]
					0.766	0.775	0.791	0.788	0.8492		[37]
									0.8897	0.4117	[40]
					0.59	0.916	0.673	0.629	0.97		[41]
		✓	✓	✓	0.72		0.78		0.902		[30]
					0.8178		0.8473	0.8294	0.9018		[31]
					0.4609				0.8357	0.4331	[35]
					0.94	0.9092	0.96	0.95	0.54	0.94	[36]
									0.8934	0.2509	[40]
					0.554	0.908	0.657	0.601	0.976		[41]
	AdaBoost (n = 2)	✓			0.749		0.685	0.637	0.618		[27]
		✓	✓			0.9024		0.8963			[28]
	XGBoost (n = 2)	✓			0.777		0.776	0.681	0.660		[27]
		✓	✓	✓	0.7154		0.8196	0.8175	0.8921		[31]
	Single-layer stacking (n = 1)	✓			0.795		0.822	0.699	0.685		[27]
	Multi-layer stacking (n = 2)	✓			0.793		0.837	0.696	0.680		[27]
									0.84		[48]
SVM	SVM (n = 6)	✓			0.746		0.92	0.656	0.639		[27]
					0.5078	0.9503			0.9052	0.4180	[32]
					0.4917		0.7992	0.5712			[39]
		✓	✓			0.9152		0.9147			[28]
								0.773			[38]
									0.7814	0.3637	[47]
	SMO (n = 2)	✓	✓	✓	0.85	0.9361	0.89	0.87	0.44	0.93	[36]
					0.92	0.98	0.96	0.98	0.63		[42]
Neighborhood-based methods	KNN (n = 7)	✓			0.5757		0.928	0.7091			[39]
		✓	✓			0.9071		0.9054			[28]
								0.719			[38]
		✓	✓	✓	0.5083				0.8835	0.4341	[35]
					0.615	0.930	0.235	0.340	0.745		[41]
					0.5008				0.8963	0.4557	[46]
						0.9149		0.5612	0.7362		[34]
	LNSM (n = 2)	✓			0.5126				0.8941	0.4491	[32]
		✓	✓	✓	0.5329				0.9091	0.4909	[45]
	INBM (n = 1)	✓	✓	✓	0.606	0.959	0.607	0.606	0.934	0.641	[44]
Regression	Logistic (n = 3)	✓			0.756		0.718	0.660	0.642		[27]
		✓	✓			0.9157		0.9115			[28]
		✓	✓	✓	0.7933		0.8014	0.7973	0.9018		[31]
	Linear (n = 1)	✓	✓	✓	0.2854				0.6724	0.2595	[35]
	Regularized (n = 1)	✓			0.3607	0.9435			0.7506	0.3015	[32]
Neural network	MLP (n = 5)	✓	✓			0.8616		0.8688			[28]
						0.7416	0.537		0.695		[29]
									0.8941	0.4165	[47]
		✓	✓	✓		0.9087		0.6031	0.7234		[34]
					0.5196				0.9003	0.4967	[35]
	RBF (n = 1)	✓	✓	✓				0.761			[38]
	RBMBM (n = 1)	✓	✓	✓	0.581	0.957	0.608	0.594	0.941	0.616	[44]
	ELM (n = 1)	✓	✓	✓				0.699			[38]
Bayes theorem	Naive Bayes (n = 3)	✓	✓			0.8528		0.8296			[28]
		✓	✓	✓	0.7682		0.8240	0.7951	0.8713		[31]
					0.377	0.919	0.431	0.402	0.7		[41]
	Bayesian network (n = 1)	✓	✓			0.8473		0.8225			[28]
Clustering	K-means (n = 1)	✓	✓	✓					0.895	0.404	[43]
	K-seeds (n = 1)	✓	✓	✓					0.894	0.404	[43]
	PAM (n = 1)	✓	✓	✓					0.895	0.399	[43]
Decision tree	Decision tree (n = 2)	✓	✓			0.9170		0.9142			[28]
		✓	✓	✓	0.2252				0.6634	0.1989	[35]
	PART (n = 1)	✓	✓			0.9192		0.9166			[28]
	RIPPER (n = 1)	✓	✓			0.9215		0.9181			[28]
Other algorithms	Sparse canonical correlation analysis (n = 1)	✓	✓						0.8230	0.3444	[47]
	Matrix decomposition model (n = 1)	✓	✓						0.920	0.59	[33]

Abbreviations: AdaBoost (Adaptive Boosting); AUC (Area under the Curve); AUPR (Area Under the Precision–Recall curve); INBM (Integrated Neighborhood-Based Method); KNN (K-Nearest Neighbor); LNSM (Linear Neighborhood Similarity Method); MLP (Multi-layer Perceptron); PAM (Partition Around Medoids); RBF (Radial Basis Function); RBMBM (Restricted Boltzmann Machine-Based Method); RIPPER (Repeated Incremental Pruning to Produce Error Reduction); SMO (Sequential Minimal Optimization); SVM (Support Vector Machine); XGBoost (eXtreme Gradient Boosting).

## Data Availability

All data represented are available in the public domain.

## Supplementary Materials

The following supporting information can be downloaded at: https://www.mdpi.com/article/10.3390/ph17060795/s1, Table S1: Search strategies in databases.
