# Supplementary material for: Machine Learning Techniques for Predicting Drug-Related Side Effects: A Scoping Review
Source: Pharmaceuticals (Basel). 2024 Jun 17;17(6):795. doi: 10.3390/ph17060795 (PMC11206653; doi:10.3390/ph17060795)
Supplement: Supplementary file 1 [file pharmaceuticals-17-00795-s001.zip › pharmaceuticals-2987043-supplementary.pdf]

Supplementary materials

Table S1. Search strategies in databases

Database	Search strategy 4	Number of records
**PubMed**	(("drug-related side effects and adverse reactions"[MeSH Terms] OR "drug side effect*"[Title/Abstract] OR "adverse drug reaction*"[Title/Abstract] OR "adverse drug event"[Title/Abstract] OR "drug toxicity"[Title/Abstract] OR "medication side effect"[Title/Abstract]) AND ("Machine Learning"[MeSH Terms] OR "Artificial intelligence"[MeSH Terms]) AND ("Forecasting" [MeSH Terms] OR "Predict*"[Title/Abstract])) AND (2013:2023[pdat]) AND (english[Filter])	387
**Scopus**	TITLE-ABS-KEY ((("drug-related side effects and adverse reactions" OR "drug side effect" OR "adverse drug reaction" OR "adverse drug event" OR "drug toxicity" OR "medication side effect") AND ("Machine Learning" OR "Artificial intelligence") AND ("Forecasting" OR "Predict"))) AND PUBYEAR > 2012 AND PUBYEAR < 2024 AND (LIMIT-TO (LANGUAGE , "English"))	612
**Web Of Science**	(("drug-related side effects and adverse reactions" OR "drug side effect" OR "adverse drug reaction" OR "adverse drug event" OR "drug toxicity" OR "medication side effect") AND ("Machine Learning" OR "Artificial intelligence") AND ("Forecasting" OR "Predict")) (Topic) AND English (Language)<br>Timespan: 2013-01-01 to 2023-12-30 (Publication Date)	212
**The Cochrane library**	(("drug-related side effects and adverse reactions" OR "drug side effect" OR "adverse drug reaction" OR "adverse drug event" OR "drug toxicity" OR "medication side effect") AND ("Machine Learning" OR "Artificial intelligence") AND ("Forecasting" OR "Predict")) in Title Abstract Keyword - with Cochrane Library publication date Between Jan 2013 and Dec 2023	0
**Ovid Medline**	(("drug-related side effects and adverse reactions" or "drug side effect" or "adverse drug reaction" or "adverse drug event" or "drug toxicity" or "medication side effect") and ("Machine Learning" or "Artificial intelligence") and ("Forecasting" or "Predict")).ti. or (("drug-related side effects and adverse reactions" or "drug side effect" or "adverse drug reaction" or "adverse drug event" or "drug toxicity" or "medication side effect") and ("Machine Learning" or "Artificial intelligence") and ("Forecasting" or "Predict")).ab. or (("drug-related side effects and adverse reactions" or "drug side effect" or "adverse drug reaction" or "adverse drug event" or "drug toxicity" or "medication side effect") and ("Machine Learning" or "Artificial intelligence") and ("Forecasting" or "Predict")).kw.<br>limit to (english language and yr="2013 - 2023")	134
**ProQuest**	title((("drug-related side effects and adverse reactions" OR "drug side effect" OR "adverse drug reaction" OR "adverse drug event" OR "drug toxicity" OR "medication side effect") AND ("Machine Learning" OR "Artificial intelligence") AND ("Forecasting" OR "Predict"))) OR abstract((("drug-related side effects and adverse reactions" OR "drug side effect" OR "adverse drug reaction" OR "adverse drug event" OR "drug toxicity" OR "medication side effect") AND ("Machine Learning" OR "Artificial intelligence") AND ("Forecasting" OR "Predict"))) OR mainsubject((("drug-related side effects and adverse reactions" OR "drug side effect" OR "adverse drug reaction" OR "adverse drug event" OR "drug toxicity" OR "medication side effect") AND ("Machine Learning" OR "Artificial intelligence") AND ("Forecasting" OR "Predict")))<br>Additional limits - Date: From January 01 2013 to December 30 2023; Language: English	55
**IEEE Xplore**	(("drug-related side effects and adverse reactions" OR "drug side effect" OR "adverse drug reaction" OR "adverse drug event" OR "drug toxicity" OR "medication side effect") AND ("Machine Learning" OR "Artificial intelligence") AND ("Forecasting" OR "Predict"))<br>Filters Applied: 2013 - 2023	57
